# Learned Attentional Strategies in Word Holistic Processing

**DOI:** 10.3390/vision8030041

**Published:** 2024-06-25

**Authors:** Paulo Ventura, Isabel Leite, Alexandre Pereira, Francisco Cruz

**Affiliations:** 1 Faculdade de Psicologia, Universidade de Lisboa, 1649-004 Lisboa, Portugal; franciscocorreiadacruz@gmail.com; 2 Centro de Investigação em Ciências Psicológicas (CICPSI), Faculdade de Psicologia, Universidade de Lisboa, 1649-013 Lisboa, Portugal; 3 Departamento de Psicologia, Universidade de Évora, 7004-516 Évora, Portugal; ijsantossilva@gmail.com; 4 Centre for Research in Applied Communication, Culture and New Technologies (CICANT), Universidade Lusófona de Humanidades e Tecnologias, 1749-024 Lisboa, Portugal; abanha@gmail.com

**Keywords:** holistic processing, word processing, learned attention, statistical learning, congruency effects

## Abstract

Previous research has shown that, like faces, words are processed either holistically or through the automatic representation of their parts combined. The automaticity assumed to underlie the holistic processing of words presupposes that individuals have a relatively low level of control over these processes. However, they may also be capable of learning from their environments whether processing words as a whole is the most efficient processing strategy—which would require at least some control over the corresponding processes. In fact, previous research supports this latter account in the context of the holistic processing of faces: when provided a task in which participants should ignore half of a stimuli (the irrelevant part) and pay selective attention to the other half (the target part), the participants become better at ignoring the irrelevant part when it is commonly misleading (i.e., this suggests a response that is different from that of the relevant part in the context of the task). In the present work, we extend these considerations to holistic word processing. Our results support a learned attentional account in the context of holistic word processing. When an irrelevant word part is systematically helpful for the judgment of a target word half, participants engage more in holistic processing (vs. when the irrelevant word half is misleading). This reflects an incidental statistical learning process in which individuals identify the irrelevant word half as either providing helpful or misleading information about the target half.

## 1. Introduction

Perceiving faces is a complex and difficult task. Facial structures exhibit a universal configuration, characterized by a shared collection of features arranged in a common structure (e.g., the eyes are positioned above the nose and the nose is positioned above the mouth). Identifying a face necessitates the rapid and precise discrimination of subtle variations in facial features (such as eye size) and their interrelationships (such as the distance between the nose and mouth) through holistic processing [1,2,3,4,5,6]. Holistic processing refers to the attentional strategy of focusing on a whole set of diagnostic facial features at once, which is developed through extensive experience with faces [7,8,9]. Processing all parts of a face object simultaneously is an effective strategy because its diagnostic features are distributed throughout the entirety of the stimulus [10]. 

The factors that contribute to the development of holistic processing, such as the requirement for distinguishing between similar objects within a category and the influence of expertise in enhancing one’s accuracy, are applicable to other visual stimuli. Indeed, holistic processing does not seem to be limited to faces, extending to other visual categories in which people may develop expertise, such as X-rays [11], chess boards [12], fingerprints [13], and cars [3]. Processing objects at a within-category level, or individuating them, seems to underpin expertise-driven holistic processing [5].

Face recognition and word recognition have traditionally been regarded as distinct research domains. In order to achieve word recognition, categorization at the basic level is necessary [14], given that words do not all have the same number and order of elements [15]. Additionally, detailed spatial relationships between letters provide little information about the identity of a word. Face and word perception have thus been described as contrasting ends of a continuum of object recognition, according to such observations (e.g., [16,17,18,19]): part-based processing for words versus holistic processing for faces. 

However, this distinction between part-based processing for words and whole-based processing for faces could be considered an over-simplification. Face recognition presents a unique cognitive challenge for humans, as it necessitates rapid and detailed computations to differentiate between highly similar faces. It has been suggested that holistic processing, which considers all components of an object simultaneously, could help overcome this difficulty [18,20]. Like face recognition, word recognition presents a comparable challenge to the human mind. In order to effectively identify words formed by arranging a restricted set of letters that share a high degree of self-similarity, readers are required to do so rapidly [21,22]. 

Wong and colleagues [22] have begun investigating word processing using the complete composite task (i.e., all parts of a visual word are fully processed when observers perform a task regarding a word part). They found evidence for stronger holistic processing as one’s proficiency with a writing system increases, such that native English readers engage more in holistic processing of (English) words than non-native English readers. 

Ventura and collaborators [23] examined whether fluent readers (as representing experts in word stimuli) displayed a word composite effect modulated by the accessibility of their orthographic lexicon. Larger word composite effects were negatively associated with the word frequency effect. Since the latter describes the extent to which high-frequency words are easier to access than low-frequency ones, it serves as a proxy for how fast the orthographic lexicon is accessed, and thus, the results from this work suggest that holistic processing increases the efficiency of word recognition in expert readers.

Consequently, proficient word recognition could be shown by the holistic processing of visual words (i.e., the obligatory encoding of/attention given to all letters of a word). The word composite effect provides evidence for this claim, as it refers to the tendency for the entire word to be considered, even though only a portion of it is required for a particular task. This is the case for logographic and alphabetic writing systems, as research [22,24,25,26] demonstrates. 

Like faces, holistic word processing is frequently assessed using a composite task, in which participants are asked to indicate whether a matched part/syllable (e.g., left) in two consecutive dissyllabic words is the same or not. In this task, participants do not need to read or process the entirety of the word as there is an irrelevant syllable (e.g., right). Interestingly, the responses provided are influenced by the presence of the irrelevant syllable. When the response suggested by the irrelevant word part matches the correct response (i.e., pertaining to the relevant syllable), their performance is better (e.g., same-response trials: LANE–LANE; different-response trials: LANE–COZY), relative to when the responses suggested by the relevant and irrelevant parts are in conflict (e.g., same-response trials: LANE–LADY, different-response trials: LANE–CONE). Just as documented for the composite face effect, this effect of congruency with words is attenuated, albeit when the syllables in the word are not aligned (e.g., one half of the word is displaced vertically, up, or down). Congruency effects that are not modulated by alignment can also be evidence of holistic processing, although sometimes they can be found in novices as well, for strategic reasons (see [27]). 

We introduced the term “attentional strategy” above. When individuals are expected to assess specific parts of a word (left or right) or a face (top or bottom) as part of a typical composite task, the inflexibility of these attentional weightings hinders their ability to concentrate on the task-relevant portion. Consequently, their selective attention seems to fail.

In a recent study, Curby and collaborators [10] examined whether learned attention would lead to holistic processing depending on whether the half that is irrelevant to a task contained useful (i.e., congruent) or deceptive (i.e., incongruent) information more often, as this would encourage the participants to consider the irrelevant information or not, respectively. In particular, the participants might learn the likelihood that the half that is irrelevant to the task comprises congruent (consistent) or incongruent (misleading) information through incidental statistical learning. If the participants realize that the region of the half that is irrelevant to the task systematically contains misleading information that hurts their performance (i.e., that is inconsistent with the relevant word half), they may be capable of modifying their face processing to reduce the amount of processing devoted to the half that is irrelevant to the task.

In their investigation, Curby and collaborators [10] explored the attentional account of holistic processing by determining whether the degree to which individuals rely on holistic face processing is influenced by how likely it is that the half of a face that is irrelevant to a task aids or hinders their performance (i.e., by being congruent or incongruent). According to an attentional account of holistic processing, the attenuation of holistic processing would occur when the probability of congruent information being present in the portion that is irrelevant to the task is low, as opposed to when this probability is high. In particular, the congruency effect should be smaller when the irrelevant information does not contribute to accurate discrimination or when the probability that the relationship between the parts that are relevant or irrelevant to the task is congruent (i.e., are identical or distinct) is low (25%) compared to when this probability is high (75%). The results supported an attentional account of holistic processing, such that holistic processing was more pronounced in the latter case (i.e., in which there is a high probability the irrelevant part is congruent).

Following the study of Curby and collaborators [10] and the previous findings suggesting that words are, like faces, visual objects processed holistically with expertise, we examined in the present study whether the composite effect of words can be affected by manipulating the probability that the portion that is irrelevant to a task will contain congruent or incongruent information. We followed closely the design of Curby and collaborators [10]. 

If analogous mechanisms—learned attentional weightings—govern the holistic processing of words in the same way that they do for faces, then the results should exhibit a similar pattern to that in Curby and collaborators [10]. In particular, the congruency effect should be diminished when the probability that the relationship between the parts of words that are relevant and irrelevant to a task is congruent (i.e., are identical or different) is low (25%) as opposed to when this probability is high (75%), that is, there would be an interaction of congruency and the proportion of congruent trials. In contrast, if learned attentional weightings are not part of word processing, we would find no evidence of an interaction of congruency and the proportion of congruent trials.

## 2. Method

### 2.1. Participants

Using MorePower 6.0.4 [28], a sample size of 26 was determined to be required to find a medium effect size (eta^2^ = 0.25) at α = 0.05 and a power of 0.8 for a 2 × 2 repeated measures ANOVA. All students enrolled in a psychology course at Departamento de Psicologia of Universidade de Évora were invited to participate. Twenty-eight students completed the two (25% congruent trials and 75% congruent trials) sessions online (cf., below). 

The study’s protocol adhered to the ethical standards enforced by the authors’ institution, namely, abiding by the Declaration of Helsinki and their country’s regulations on research in psychology, and the study was granted approval by their institution’s ethics committee. Participation was contingent on participants agreeing to do so after reading an informed consent form.

### 2.2. Material

The stimuli were 24 quartets of four-letter (consonant–vowel.consonant–vowel) CV.CV disyllabic Portuguese words (Appendix A). We manipulated the chance of the relationship between the task-relevant and task-irrelevant components being congruent (i.e., both being the same or different). In one condition, it was high, with 75% of the components being congruent in these trials, and in the other condition, it was low, with only 25% of the components being congruent in these trials.

Each word was divided into the left and right halves by a vertical line. Quartets were created by selecting four syllables (two for each syllable in a word) and exchanging them such that each of the two initial syllables was paired with one of the two second syllables. Thus, quartets comprised four words resulting from the orthogonal manipulation of response (same; different) and congruency (congruent; incongruent): for example, caro, pule, cale, puro. By including each word in a quartet as a study and as a test stimulus, syllables worked as distractors in both congruent and incongruent trials.

### 2.3. Procedure 

**Figure 1:**
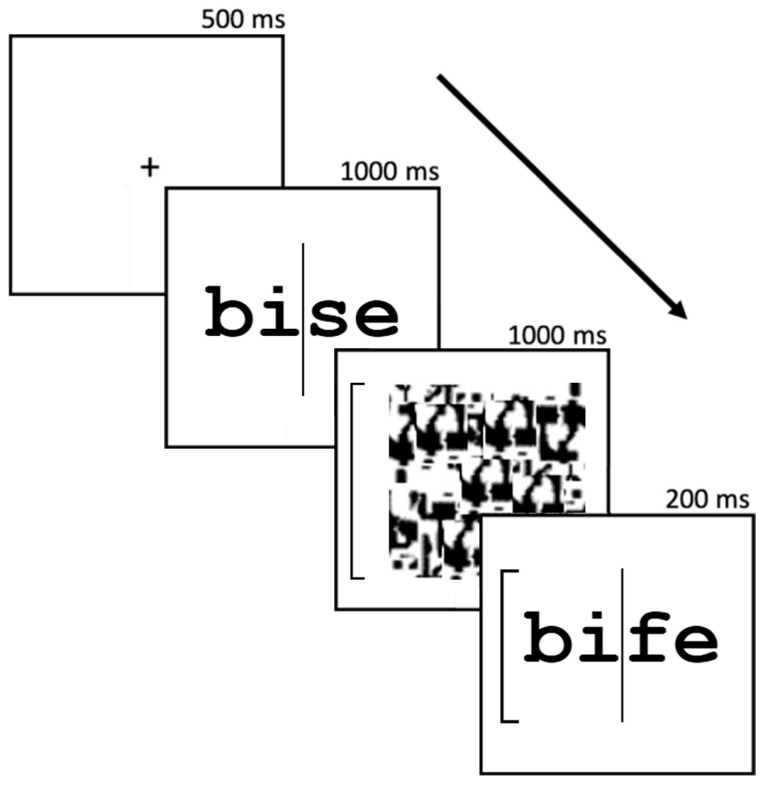
Trial structure in the composite word task.

We followed closely the procedure of Curby and collaborators [10], cf. Figure 1. Participants performed a complete version of the composite task. Following the presentation of a fixation cross in the center of the screen for 500 ms, participants saw the first word, which was presented for 1000 ms. Then, they saw a textured pattern serving as a mask for 1000 ms, accompanied by a bracket either on its left or right which indicated which half of the word participants had to base their judgment on (i.e., the task-relevant half). Finally, they were presented the second word for 200 ms and the same bracketed cue. Participants then answered whether the cued half was the same in the two words they were presented with by pressing two different keys (i.e., for same and different responses). Participants were provided feedback if they either answered incorrectly or if they failed to provide an answer within 2500 ms of the removal of the second word. Feedback was displayed for 500 ms, followed by a fixation cross for 1000 ms, before the next trial started.

Before the experimental task started, participants were offered 32 practice trials: 16 congruent and 16 incongruent. The task that followed was composed of six blocks of 64 trials, for a total of 384 trials, and participants were offered a break between blocks. Participants either saw 96 congruent (and 288 incongruent) trials or 288 congruent (and 96 incongruent) trials, depending on the session (i.e., for 25% or 75% congruent trials). The experiment was run online using EPrime Go 1.0.

## 3. Results

**Figure 2:**
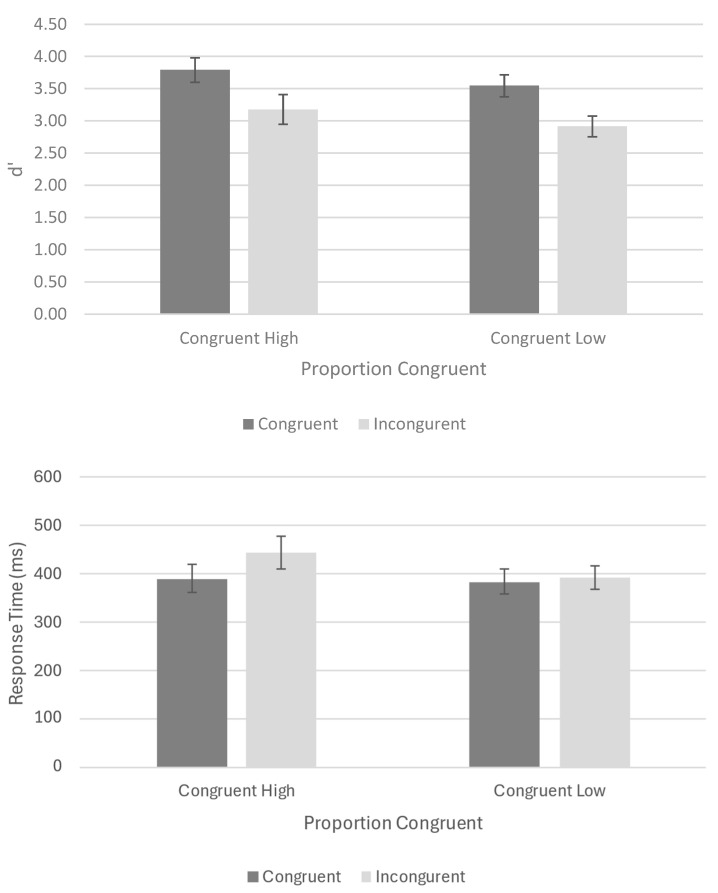
Mean sensitivity (i.e., d’; **top**) and mean response time (i.e., ms; **bottom**) for the congruent and incongruent trials under conditions of high or low proportions of congruency.

The sensitivity scores (d’) and mean response time (RT; ms) for each condition are presented in Figure 2.

### 3.1. Sensitivity 

The d’ was calculated by subtracting the z-score for false alarms (1-Hit or incorrect “same” responses) from the z-score for hits (here, correct “different” responses).

A two (congruency; congruent, incongruent) × two (proportion congruent: high (75% congruent/25% incongruent) or low (25% congruent/75% incongruent)) repeated measures ANOVA was performed on these data. The proportion of congruent trials had no effect (F(1, 27) = 2.3, *p* = 0.14, ηp^2^ = 0.08). There was an effect of congruency (F(1, 27) = 35.27, *p* ≤ 0.0001, ηp^2^ = 0.57), such that the sensitivity was higher when the irrelevant word half was congruent with the relevant one (vs. incongruent trials). This congruency effect did not, however, interact with the proportion of congruent trials (F < 1).

### 3.2. Response Time (RT) 

A two (congruency; congruent, incongruent) × two (proportion congruent: high (75% congruent/25% incongruent) or low (25% congruent/75% incongruent)) repeated measures ANOVA was performed on the RT data. The analysis revealed there was an effect of congruency (F(1, 27) = 22.93, *p* ≤ 0.0001, ηp^2^ = 0.46), with faster RTs in the congruent trials than those in the incongruent trials. The proportion of congruent trials had no effect (F(1, 27) = 2.53, *p* = 0.12, ηp^2^ = 0.09). Still, congruency and the proportion of congruent trials interacted (F(1, 27) = 14.78, *p* < 0.001, ηp^2^ = 0.36), with reaction times being more discrepant between the congruent and incongruent trials when there was a high (in comparison to low) proportion of congruent trials.

## 4. Discussion

The present research shows that manipulating the frequency of (in)congruent trials influences task performance, namely, the emergence of holistic processing. The actual congruency of the irrelevant word part interacted with the proportion of congruent irrelevant parts in the task, such that the former effect was larger when most of the trials provided congruent information (in terms of RT data). The influence of the congruency probability on the congruency effect aligns with the conclusions drawn from learned attentional accounts of holistic processing: in a high-congruency condition, participants acquire greater benefits from congruent trials compared to in a low-congruency condition [10].

Recently, holistic word processing has been framed as the result of acquired expertise of word stimuli (e.g., [14,29,30]). Accurate and quick word recognition, with a fast recruitment of visual word representations that begin phonological and representational processes, depends on the swift identification of individual letters and their relative positioning. In order to achieve efficient word recognition, we employ holistic processing. Holistic processing may be framed under the concept of inflexible attentional weightings to letters that are developed through experience. Our findings indicate that, similar to the holistic processing of faces, the context of a task influences at least a part of the holistic processing of words. Presumably, this is achieved through the ability of participants to learn, through experience, to shape their attention in a manner that is more suitable for the current task.

Thus, as people develop reading capabilities, they are essentially developing this ability to quickly discriminate letters in strings composed by elements that resemble one another [21,22]. If words are objects of expertise, like faces, and indeed holistic processing is brought about by acquiring expertise, it should follow that holistic processing emerges for words in expert word readers. This holistic strategy is optimal in word processing because accurate identification relies on the information spread across a word. This was the case of the effect of the proportion of congruent trials in the present study.

However, the extent to which other information embedded in a word may contribute to word holistic processing is an open question. Ventura and collaborators [31] explored the role of sublexical properties—specifically bigram transition probabilities—in this processing strategy. They used a composite task and four-letter disyllabic words, in which two of the bigrams reinforce the cohesiveness of each syllable and one of the bigrams reinforces the cohesiveness between the syllables. They found preliminary evidence of the role of sublexical properties in word holistic processing.

Another study showing the influence of linguistic factors on holistic processing was that by Ventura and collaborators [32]. Given the linguistic nature of written words, Ventura and collaborators [32] expected phonology to influence the word composite effect. As in the present work, participants were presented with a word composite task, in which they had to decide whether two syllables in sequentially presented words were the same or different. Importantly, the word pairs could be consistent or inconsistent in their phonology (i.e., with orthographies that always produce the same sound or that can be pronounced differently). Whether the word was phonologically consistent or not was crucial to the word composite effect, such that it was only found when the orthographic representations only mapped onto one phonological representation. This study suggests that lexical properties, such as a word’s possible phonological representation, influence holistic processing.

Thus, words are a linguistic entity, and their holistic processing is influenced by linguistic factors. But it is undeniable that the holistic processing of words shares many properties with the holistic processing of faces. In the present study, when the participants were required to evaluate a specific portion (left or right) of a word in the context of a typical composite task, the inflexible nature of their attentional weightings caused difficulties focusing on the portion that was relevant to the task when the proportion of congruent trials was high. Their attention was spread across the entire word. This gave rise to an apparent failure of their selective attention. When adopting a distributed attentional strategy is suboptimal or prone to decreasing their performance (in the 25% congruent trials), the participants processed words less holistically.

One limitation of our work is that it involved an alphabetic system of writing (i.e., in which each symbol corresponds to a spoken sound). Further work could be conducted with logographic systems of writing, in which symbols represent whole words or more complex phoneme groupings. Note that previous research has found holistic word processing to extend to logographic scripts [24,26], and thus, applying a similar paradigm to these would further allow for establishing the commonalities of holistic word processing underlying different writing systems and/or set the boundaries of such commonalities instead.

In conclusion, this study provides evidence that the holistic processing of words can be modulated by the context of a task. These findings add to the previous body of literature by bolstering our knowledge about the extent to which the holistic processing of words is sensitive to contextual information and/or incidental statistical learning.

## Data Availability

Data pertaining to this study are available at: osf (DOI 10.17605/OSF.IO/4F25C).

## Appendix A. Stimuli Used in the Experiment

**Table A1:** Quartets for high proportion of congruency (75% congruent/25% incongruent).

Set	Words			
1	BIFE (steak)	BICO (beak)	SAFE (get way)	SACO (bag)
2	BODE (goat)	BOGA (bogue)	RUDE (rude)	RUGA (wrinkle)
3	BULE (teapot)	BUDA (buddha)	ROLE (roll)	RODA (wheel)
4	DOSE (dose)	DOCA (dock)	BISE (encore)	BICA (spout)
5	DURE (last)	DUNA (dune)	MIRE (aim)	MINA (mine)
6	FIGO (fig)	FITA (ribbon)	NEGO (I deny)	NETA (granddaughter)
7	LISA (smooth)	LIGO (care)	PESA (weighs)	PEGO (I catch)
8	MEGA (mega)	MERO (mere)	FIGA (fig)	FIRO (I hurt)
9	PUDE (I could)	PUXA (pull)	LIDE (deal)	LIXA (sandpaper)
10	RIJA (tough)	RIME (rime)	FUME (smoke)	FUJA (run away)
11	SINA (lot)	SIGO (following)	RENA (reindeer)	REGO (gully)
12	VICE (vice)	VIGA (beam)	ROCE (rook)	ROGA (entreats)
13	BASE (base)	BAFO (breath)	PISE (tread)	PIFO (drunkness)
14	CUME (top)	CUJA (whose)	SOME (add)	SOJA (soy)
15	DIGA (say)	DITO (said)	NEGA (denies)	NETO (grandson)
16	FASE (phase)	FARO (flair)	VISE (aim)	VIRO (turn)
17	FOLE (bellows)	FORA (outside)	PULE (jump)	PURA (pure)
18	LEVA (takes)	LEGO (lego)	DIVA (diva)	DIGO (I say)
19	LIMA (lime)	LISO (smooth)	TEMA (theme)	TESO (stiff)
20	LUTE (fight)	LUVA (glove)	NOTE (notice)	NOVA (new)
21	REGA (irrigation)	REMO (rowing)	LIGA (alloy)	LIMO (slime)
22	TINA (tub)	TIPO (type)	CENA (scene)	CEPO (block)
23	TIVE (had)	TIRA (strip)	NOVE (nine)	NORA (daughter-in-law)
24	VIME (rattan)	VILA (town)	COME (eat)	COLA (glue)

**Table A2:** Quartets for low proportion of congruency (25% congruent/75% incongruent).

Set	Words			
1	FETO (fetus)	MIRA (aim)	FERA (beast)	MITO (myth)
2	PELO (for the)	SIGA (follow)	PEGA (handle)	SILO (garner)
3	FILA (row)	VETO (negative)	FITO (regard)	VELA (candle)
4	RISO (laughter)	PETA (lie)	RITA (Rita)	PESO (weght)
5	CERA (wax)	LIDO (read)	CEDO (early)	LIRA (lyre)
6	LUME (fire)	TOPA (check it out)	LUPA (magifying glass)	TOME (take)
7	CEDO (early)	TIPA (guy)	CEPA (strain)	TIDO (had)
8	BOLA (ball)	PUFE (pouf)	BOFE (lung)	PULA (jump)
9	TIDO (had)	GARE (station)	TIRE (remove)	GADO (cattle)
10	RICA (rich)	TEJO (Tagus river)	RIJO (stiff)	TECA (teak)
11	ZERO (zero)	GILA (gila)	ZELA (caretaker)	GIRE (rotate)
12	ROLA (dove)	MUDE (change)	RODE (rotate)	MULA (mule)
13	COTA (quota)	JURE (swear)	CORE (blush)	JUTA (jute)
14	RITO (rite)	MEXA (mix)	RIXA (feud)	METO (incase)
15	TIRE (remove)	SAPO (frog)	TIFO (typhus)	SARE (heal)
16	GIRE (rotate)	FULA (furious)	GILA (gila)	FURE (pierce)
17	CALE (shut up)	VIRO (I turn)	CARO (expensive)	VICE (vice)
18	CANO (pipe)	FITE (regard)	CATE (pick up)	FINO (thin)
19	CARO (expensive)	MEÇO (I measure)	CAÇO (hunt)	MERO (mere)
20	CASO (case)	VIÇO (freshness)	CAÇO (hunt)	VISO (aim)
21	CENA (cene)	TIPO (type)	CEPO (log)	TINA (trough)
22	CIMA (top)	DOTE (dowry)	CITE (quote)	DOMA (tame)
23	COMI (I ate)	GERA (generates)	CORA (flush)	GEMI (I moaned)
24	CATE (pick up)	DIVO (divo)	CAVO (dig)	DITE (dictate)
